# Corpus-level behavioral intensity in human–AI conversations

**DOI:** 10.3389/frai.2026.1914081

**Published:** 2026-09-18

**Authors:** Aracely Mera-Navarrete, Solange Revelo, Jefferson Beltrán-Morales, William Villegas-Ch

**Affiliations:** 1 Escuela de Comunicación, Facultad de Jurisprudencia, Ciencias Sociales y Humanidades Andrés F. Córdova, Universidad Internacional del Ecuador, Quito, Ecuador; 2 Unidad Educativa Particular San Gabriel, Quito, Ecuador; 3 Facultad de Ingeniería y Ciencias Aplicadas, Universidad Central del Ecuador, Quito, Ecuador; 4 Escuela de Ingeniería en Ciberseguridad, Facultad de Ingeniería y Ciencias Aplicadas, Universidad de Las Américas, Quito, Ecuador

**Keywords:** behavioral intensity, Chatbot datasets, corpus-level analysis, human-AI interaction, large language models

## Abstract

Conversational generative models are increasingly used to produce, transform, and revise information through multi-turn exchanges. However, large human–AI datasets are still commonly analyzed through performance, preference, or content lenses, leaving conversation-level behavioral structure less operationalized. This study proposes a transparent rule-based framework for estimating persistence, delegation-related lexical patterns, and refinement-related lexical markers without inferring psychological dependence. LMSYS-Chat-1M and WildChat were analyzed as primary conversational corpora, while Chatbot Arena was included as a structurally conditional A/B comparator. Across 1,879,085 valid analytical records, we calculated IPF, CDR, SRS, and the Conversational Behavioral Intensity Index (CBII), together with an equal-weight control. WildChat showed the highest mean CBII (0.2463), followed by LMSYS-Chat-1M (0.2123) and Chatbot Arena (0.1762). This ordering was stable under language controls, IPF thresholds of 5, 10, and 20 user turns, and question-level analysis of Chatbot Arena. Pairwise effect sizes were small to moderate, with the largest contrast between WildChat and Chatbot Arena (Cohen's *d* = 0.363).

## 1 Introduction

Conversational generative models have transformed human–AI interaction into a daily practice of searching for, producing, and revising information. Unlike traditional predictive systems, large language models operate through sequential exchanges in which users consult the system, formulate explicit task requests, ask for reformulations, correct outputs, and extend the interaction until a useful response is obtained. This dynamic has shifted part of the debate from model accuracy alone to the observable structure of use, including persistence, delegation-related request patterns, and revision-oriented lexical signals. Recent literature highlights the need to study these relationships from a user-centered perspective supported by behavioral records, especially when conversational systems mediate decision-making, learning, persuasion, or informational support (Sun et al., 2023; Jiang et al., 2024; Vaccaro et al., 2024).

Measuring these patterns remains methodologically challenging. Studies on overtrust, explainability, and human–AI collaboration have shown that users may rely on automated recommendations under specific task conditions, and that cognitive forcing or explanation design can alter such reliance (Vasconcelos et al., 2023; de Jong et al., 2025). In parallel, research on social and companion chatbots has documented sustained interaction, communicative attachment, and emotionally charged uses of conversational agents (Pentina et al., 2023; Kim et al., 2025; Jocher and Verwiebe, 2026). These approaches provide rich evidence about attitudes, trust, social meaning, or task outcomes, but they are often based on surveys, controlled experiments, platform-specific case studies, or thematic interpretation. Large open conversational corpora require a complementary descriptive layer: indicators that can be calculated directly from structural and textual traces, audited without additional models, and interpreted without assigning psychological states to individual users.

This work addresses that need by using a compact framework of behavioral indicators calculated at the level of the entire conversation. The proposal does not aim to diagnose psychological dependence, measure addiction, or estimate the causal effects of AI use. Its operational objective is to evaluate whether transparent textual and structural signals capture differences between open corpora of human–AI interaction. To this end, three recent sources are used: LMSYS-Chat-1M, WildChat, and Chatbot Arena. LMSYS-Chat-1M and WildChat are considered the main conversational datasets, while Chatbot Arena is included as a conditional comparator because its native structure is an A/B evaluation design rather than an independent conversational corpus.

The unit of analysis is the complete conversation. Each record was normalized to extract the identifier, dataset, model, language, user and assistant turns, and aggregated text. Conversations without a user or assistant, with fewer than two turns, with empty text, with a total length of less than 20 characters, or with duplicate identifiers were excluded. Three components were calculated for the valid conversations: interaction persistence factor (IPF), conversational delegation ratio (CDR), and self-refinement score (SRS). IPF measures persistence through user turns; CDR estimates the proportion of user messages containing delegation-related lexical markers; and SRS estimates the proportion of user messages containing correction, modification, or refinement markers. These components are integrated into the conversational behavioral intensity index (CBII) and an equally weighted sensitivity version (*CBII*_*equal*_).

Motivated by this, this work makes three main contributions. First, it proposes a lightweight set of indicators based on conversational persistence, delegation-related lexical markers, and refinement-related lexical markers, designed to be transparent and reproducible from open conversational datasets. Second, it performs a cross-dataset empirical comparison using two primary conversational corpora and one structurally conditional comparator. Third, it incorporates robustness evidence for large-scale comparison, including language controls, IPF threshold sensitivity, pairwise effect sizes with bootstrap confidence intervals, question-level analysis of Chatbot Arena, exact first-prompt overlap checks, and single-turn structure controls.

The results show that the framework differentiates observable corpus-level patterns across heterogeneous human–AI datasets. After filtering, 990,758 valid records were retained from LMSYS-Chat-1M and 822,408 from WildChat; Chatbot Arena contributed 65,919 valid A/B-derived analytical records and was additionally examined through a question-level control. WildChat presented the highest mean CBII (0.2463), followed by LMSYS-Chat-1M (0.2123) and Chatbot Arena (0.1762). The ordering was maintained using *CBII*_*equal*_, under English-only and English + Spanish controls, and across IPF saturation thresholds of 5, 10, and 20 user turns. Pairwise effect sizes were small to moderate, and the largest contrast was observed between WildChat and Chatbot Arena [mean difference = 0.0701; 95% bootstrap CI (0.0698, 0.0746); Cohen's *d* = 0.363]. These findings indicate that the proposed indicators are useful for corpus-level behavioral characterization, while their interpretation remains descriptive and non-diagnostic.

The manuscript proceeds from operational definition to empirical evaluation. The methodological section defines the conversational unit, the normalization rules, the three behavioral indicators, and the robustness controls. The results section then evaluates analytical coverage, component behavior, distributional structure, language sensitivity, effect magnitude, and structural controls. The final sections interpret the contribution, delimit the scope of inference, and identify validation needs for subsequent work.

## 2 Literature review

Recent work on LLM evaluation has emphasized that model assessment cannot rely only on aggregate accuracy or leaderboard performance. Evaluation frameworks now distinguish task performance, robustness, calibration, reliability, safety, and human-facing behavior, while also warning that inconsistent evaluation designs can produce unstable conclusions (Chang et al., 2024). Related evidence shows that larger and more instruction-following models may still become less reliable under specific prompting conditions, making user-facing interaction traces important for understanding how conversational systems are used beyond benchmark settings (Zhou et al., 2024). In this context, LLM-as-a-judge methods provide scalable semantic assessment, but they introduce model-dependent decisions, prompt sensitivity, and potential evaluator bias (Gu et al., 2026).

A second line of research focuses on trust, reliance, and overreliance in AI-supported decisions. Experimental evidence indicates that people may rely on AI advice even when such reliance generates decision costs, especially under uncertainty (Klingbeil et al., 2024). Studies of LLM explanations further show that users can overestimate what models know, and that explanation length or uncertainty communication affects perceived accuracy (Steyvers et al., 2025). Broader reviews of trust in AI describe trust as a multidimensional construct shaped by system performance, transparency, user expertise, and context (Afroogh et al., 2024). However, these works primarily examine calibrated trust, advice acceptance, or decision outcomes; they do not provide a direct corpus-level mechanism for comparing persistence, explicit request language, and revision markers across large conversational logs.

Empirical studies with LLM-generated recommendations and qualitative analysis clarify why this distinction matters. Users do not necessarily trust LLM recommendations more than other AI-sourced snippets, suggesting that conversational presentation alone does not imply higher epistemic reliance (McGrath et al., 2024). At the same time, recent comparisons between LLMs and human analysts in qualitative research show that LLMs can support summarization, coding, and theme identification, but their agreement with human interpretation varies across tasks, particularly when contextual, inferential, or affective nuance is required (Castellanos et al., 2025; Mehta et al., 2025; Hill et al., 2026). These findings support a conservative methodological position: behavioral traces should not be automatically translated into psychological states, and semantic interpretation should not be delegated entirely to opaque model-based evaluators.

The gap addressed here is therefore narrower and methodological. Existing studies provide strong evidence on LLM evaluation, trust calibration, overreliance, and model-assisted textual interpretation, but they do not offer a lightweight, reproducible index for describing observable conversation-level intensity in open human–AI datasets. CBII is designed to occupy this intermediate layer. It does not replace human validation or semantic analysis; instead, it operationalizes auditable signals that can be calculated before more expensive annotation, embedding, or LLM-judge procedures are applied.

## 3 Materials and methods

### 3.1 Data sources and conversational unit

The study used three open datasets of human–AI conversations: LMSYS-Chat-1M, WildChat, and Chatbot Arena. LMSYS-Chat-1M and WildChat were treated as the primary conversational sources because they contain conversation identifiers, models, language, and nested lists of turns with user and assistant roles. Chatbot Arena was included as a conditional comparator because its native unit is an A/B comparison of model responses. The analysis therefore distinguishes between the A/B candidate-conversation transformation and a stricter question-level control based on question_id, preventing the user prompt from being interpreted as two independent user-side observations.

The unit of analysis was the complete conversation, defined as an ordered sequence of messages with a role and textual content. For each conversation, the following were preserved: identifier, dataset, model, language, total number of turns, number of user turns, number of assistant turns, aggregated user text, aggregated assistant text, and full text. Transparent behavioral indicators were calculated for valid conversations, followed by descriptive analysis, internal-structure correlations, language controls, sensitivity assessment, effect-size estimation, overlap checks, and single-turn structure controls.

### 3.2 Canonical normalization and validity rules

Each dataset was normalized separately before any comparison. In LMSYS-Chat-1M, conversation_id was used; in WildChat, conversation_hash; and in the Chatbot Arena A/B transformation, derived identifiers were constructed using question_id::a and question_id::b. Chatbot Arena was also recalculated at the question_id level as a structural control. Turns were extracted from the nested conversation lists and separated according to user and assistant roles. This decision avoids mixing datasets before resolving structural differences and maintains traceability between raw records, processed Parquet data, and final tables.

A conversation was considered valid only if it simultaneously met the following formal conditions: *T*_*i*_≥2, *U*_*i*_>0, *A*_*i*_>0, *L*_*i*_≥20, and *dup*_*i*_ = 0, where *T*_*i*_ is the total number of turns, *U*_*i*_ is the number of user turns, *A*_*i*_ is the number of assistant turns, *L*_*i*_ is the total text length, and *dup*_*i*_ is the duplication indicator associated with each identifier within the dataset. Conversations with empty text, fewer than 20 characters, no user or assistant, a low number of turns, or duplicates were excluded.

The sequence represented in Figure 1 formalizes the validity rules as a reproducible measurement pipeline: structural normalization is completed before behavioral scoring, and robustness controls are applied after index construction. This organization separates dataset-specific formatting decisions from the substantive comparison of conversational behavior.

**Figure 1:**
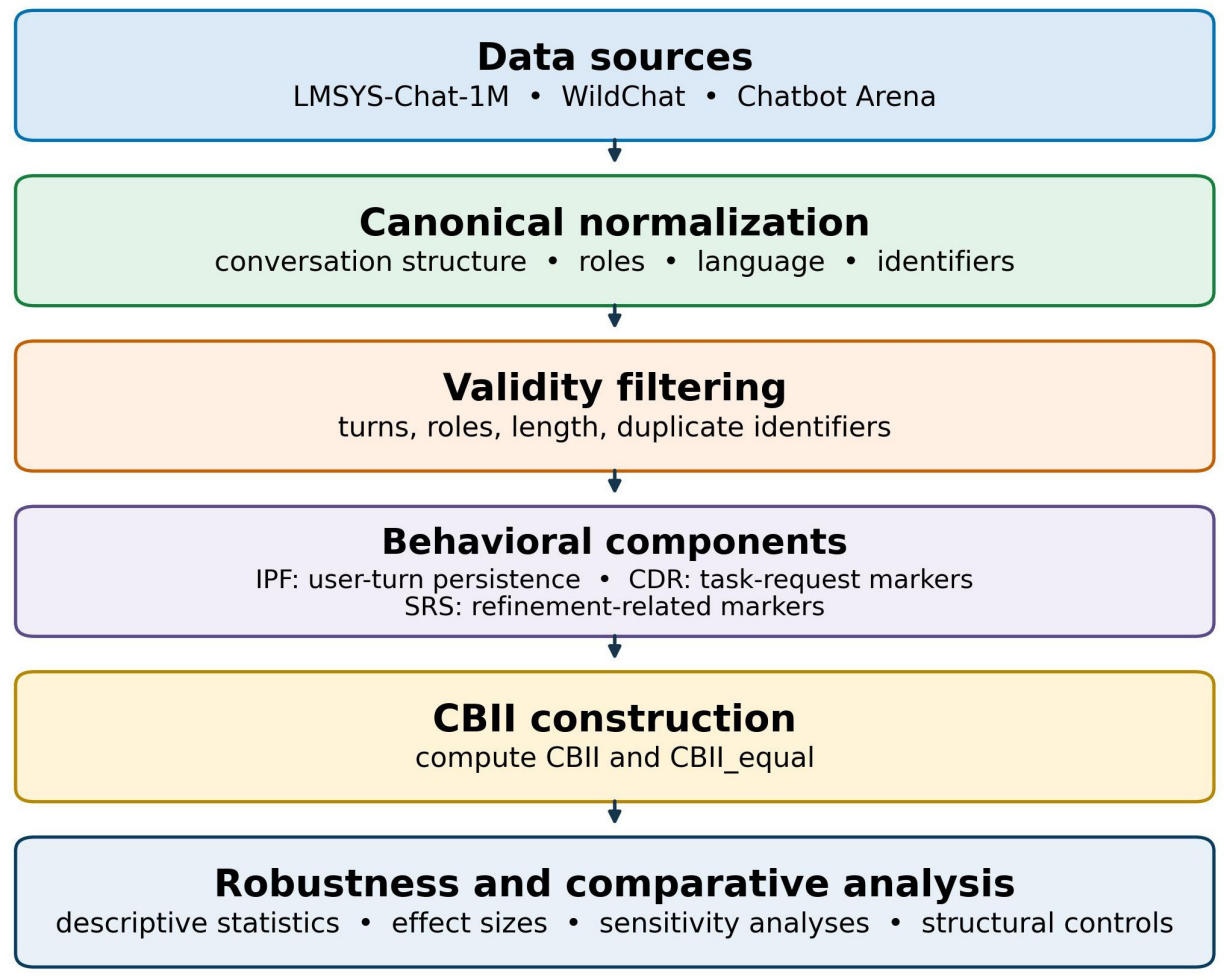
Processing flow used to calculate CBII at the conversation level and to conduct robustness controls. The framework estimates observable behavioral signals rather than psychological dependence.

### 3.3 Operationalization of behavioral indicators

For each valid conversation *i*, three components were calculated. Let *U*_*i*_ denote the number of user turns, *D*_*i*_ the number of user messages containing at least one delegation-related marker, and *R*_*i*_ the number of user messages containing at least one refinement-related marker. Markers were applied exclusively to user messages, not to assistant responses, to preserve the user-side focus of the analysis.

Delegation-related markers were identified through a predefined dictionary of task-oriented expressions, including terms such as *write, generate, create, solve, analyze, summarize, translate, explain*, and *give me*, together with Spanish equivalents. Refinement-related markers were identified through a separate dictionary containing revision expressions such as *improve, better, change, modify, rewrite, expand, shorten, fix, correct*, and *adjust*. These dictionaries are treated as transparent rule-based heuristics for corpus-level screening, not as validated psychometric instruments. Because the markers can appear in the first user message, SRS is interpreted as a lexical refinement indicator rather than as direct proof of sequence-based revision. The complete marker dictionaries and implementation details are provided in the reproducibility package to support direct audit and replication.

The first component, termed the *Interaction Persistence Factor* (IPF), quantifies conversational persistence through the number of user turns. It is defined as:


$$\begin{array}{l} IPF_{i}=\min\left(\frac{U_{i}}{10},1\right) \end{array}$$ (1)

Equation 1 normalizes conversational persistence to the interval [0, 1]. A saturation threshold of 10 user turns was adopted as a conservative normalization mechanism to prevent exceptionally long conversations from disproportionately influencing the metric while preserving meaningful variation across most interactions. Consequently, conversations with ten or more user turns receive the maximum persistence value.

The second component, termed the *Conversational Delegation Ratio* (CDR), estimates the proportion of user messages containing explicit task-request markers:


$$\begin{array}{l} CDR_{i}=\frac{D_{i}}{U_{i}} \end{array}$$ (2)

Equation 2 estimates the relative frequency of delegation-related lexical patterns within a conversation. Higher values indicate that a larger proportion of user messages contain explicit request markers associated with content generation, problem solving, explanation, translation, or similar task-oriented prompts.

The third component, termed the *Self-Refinement Score* (SRS), detects lexical signals associated with correction, modification, or refinement:


$$\begin{array}{l} SRS_{i}=\frac{R_{i}}{U_{i}} \end{array}$$ (3)

Equation 3 estimates the proportion of user messages containing correction, modification, or refinement markers. Higher values indicate greater presence of revision-related lexical patterns, but they do not establish that the user adjusted a prior request unless the conversational sequence is inspected at a finer semantic level.

All three indicators were normalized to the interval [0, 1], allowing direct comparison despite differences in their operational interpretation. This bounded representation facilitates the subsequent construction of the composite index while preserving the separate contribution of persistence, delegation-related markers, and refinement-related markers.

### 3.4 Composite index, internal structure, and robustness controls

CBII aggregates persistence, delegation-related lexical markers, and refinement-related lexical markers as a bounded conversation-level descriptor of observable interaction intensity. The index does not replace the individual indicators or infer latent psychological dependence; it provides a compact representation of how the three operational dimensions co-occur within each conversation.

The Conversational Behavioral Intensity Index (CBII) was defined as a weighted linear combination of the three components:


$$\begin{array}{l} CBII_{i}=0.40IPF_{i}+0.35CDR_{i}+0.25SRS_{i} \end{array}$$ (4)

Equation 4 implements an operational weighting scheme that places greater emphasis on conversational persistence and explicit task-request language. SRS was retained as a complementary dimension reflecting revision-related lexical evidence. These weights were not empirically optimized and do not represent the psychological importance of the components.

To assess the robustness of the weighting strategy, a sensitivity version of the index was also calculated using equal weights:


$$\begin{array}{l} CBII_{i}^{equal}=\frac{IPF_{i}+CDR_{i}+SRS_{i}}{3} \end{array}$$ (5)

Equation 5 serves as a control formulation in which persistence, delegation-related markers, and refinement-related markers contribute equally to the final score. Comparing CBII and *CBII*^*equal*^ allows the evaluation of whether observed corpus-level patterns depend critically on the proposed weighting structure or remain stable under alternative aggregation assumptions.

The descriptive analysis produced a summary matrix for each dataset, including the number of raw and valid conversations, the percentage of valid records, and the mean values of conversational turns, IPF, CDR, SRS, CBII, and *CBII*^*equal*^. The distribution of CBII was visualized through smoothed density profiles and empirical cumulative distribution functions, while component structure was represented through mean values and high-score rates. Smoothed density profiles were generated from 80-bin normalized histograms using a five-bin moving-average kernel on the bin centers; the empirical cumulative distribution was calculated directly from sorted CBII values. The high-score threshold of ≥0.5 was used only as a descriptive visual cutoff to compare component prevalence across datasets; it is not a clinical, statistical, or externally validated threshold.

Relationships among indicators were examined using Pearson and Spearman correlation coefficients across the aggregated corpus. Because CBII is a linear combination of IPF, CDR, and SRS, correlations between the components and CBII were interpreted as internal-structure diagnostics rather than independent validation evidence. The primary correlations of interest were IPF–CDR, IPF–SRS, IPF–CBII, CDR–SRS, CDR–CBII, and SRS–CBII.

Sensitivity assessment was performed by comparing CBII and *CBII*^*equal*^ through mean differences, mean absolute differences, correlation analysis, and ranking stability across datasets. Additional robustness checks recalculated the persistence component using IPF saturation thresholds of 5, 10, and 20 user turns; compared global results with English-only and English + Spanish subsets; estimated pairwise mean differences with percentile-based 95% bootstrap confidence intervals, Cohen's *d*, and Cliff's delta; recalculated Chatbot Arena at the question_id level; and checked exact cross-dataset overlap using SHA-256 hashes of normalized first user rompts.

The analytical pipeline was also examined through computational consistency checks. These checks verified that all indicators remained within the interval [0, 1], that every valid conversation contained at least one user turn, that no duplicate identifiers persisted after filtering, and that the counts obtained from the processed Parquet datasets matched the reported analytical totals.

### 3.5 Implementation and reproducibility

The analytical pipeline was executed using local Parquet files derived from the public Hugging Face datasets listed in the Data Availability Statement. Dataset provenance was documented on 30 July 2026, including the source URL, analytical unit, raw and valid record counts, and redistribution restrictions. The pipeline was implemented in Python using pandas, numpy, pyarrow, regular expressions, hashlib, and matplotlib. Textual normalization converted role labels and language labels to lowercase, stripped empty content, concatenated user-side and assistant-side text separately, and preserved the full conversation text for validity checks. Dictionary matches were performed on user messages only using case-insensitive regular expressions built from escaped marker strings. Exact overlap controls used SHA-256 hashes of normalized first user prompts after lowercasing and whitespace normalization. Percentile-based confidence intervals for pairwise mean CBII differences were estimated with 1,000 bootstrap replications, random seed 42, and a maximum of 50,000 sampled observations per group. The reproducibility package contains the analysis scripts, full English and Spanish marker dictionaries, aggregated analytical tables, computational consistency outputs, and dataset provenance documentation.

## 4 Results

### 4.1 Usefulness of the datasets and analytical coverage

Table 1 summarizes the analytical coverage obtained after applying the filtering criteria. The criteria retained high coverage in all three sources, but the resulting units were not methodologically equivalent. LMSYS-Chat-1M and WildChat provided 990,758 and 822,408 valid conversation-level records, respectively, making them the two primary open conversational corpora. Chatbot Arena retained 65,919 valid A/B-derived candidate conversations; however, its native paired-comparison design means that the same user prompt can be associated with two model responses. For this reason, Arena is interpreted as a conditional comparator rather than as a fully equivalent conversational corpus in this first coverage assessment.

**Table 1:** Dataset quality and analytical coverage after filtering.

Dataset	Raw records	Valid records	Valid (%)	Unit	Analytical role
LMSYS-Chat-1M	1,000,000	990,758	99.08	Conversation	Primary open conversational corpus
WildChat	837,989	822,408	98.14	Conversation	Primary open conversational corpus
Chatbot Arena	66,000	65,919	99.88	A/B candidate	Conditional comparator with question-level control

The main exclusions varied across the datasets. In LMSYS-Chat-1M, short conversations and instances without an assistant response predominated. In WildChat, the largest adjustment was associated with duplicate conversational identifiers. Chatbot Arena showed minimal filtering losses, but its native paired-comparison structure requires caution: the A/B transformation is useful for comparing response candidates, whereas the question-level control is more appropriate for user-side behavioral interpretation.

### 4.2 Descriptive matrix of behavioral indicators

Table 2 establishes the empirical ordering of the three corpora at the component and composite levels. WildChat obtained the highest mean values in all three components: IPF (0.2210), CDR (0.3773), and SRS (0.1035). This dataset also exhibited the highest mean CBII (0.2463) and the highest *CBII*_*equal*_ (0.2339). LMSYS-Chat-1M showed an intermediate profile, with a mean CBII of 0.2123 and a mean *CBII*_*equal*_ of 0.2012. Chatbot Arena obtained the lowest mean CBII (0.1762) and mean *CBII*_*equal*_ (0.1689), consistent with its shorter and comparison-oriented conversational structure.

**Table 2:** Descriptive matrix of behavioral indicators at the conversation level.

Dataset	IPF	CDR	SRS	CBII	CBII_*eq*_	Mean turns
LMSYS-Chat-1M	0.1915	0.3271	0.0849	0.2123	0.2012	4.0247
WildChat	0.2210	0.3773	0.1035	0.2463	0.2339	4.7120
Chatbot Arena	0.1190	0.3168	0.0709	0.1762	0.1689	2.3823

The proposed framework represents three complementary operational dimensions rather than a single behavioral construct. CDR showed the highest mean values across all datasets, suggesting that explicit task-request markers are frequent in open conversations between humans and AI. SRS had lower values than CDR, indicating that refinement-related lexical markers occur less frequently than delegation-related request markers. IPF varied across datasets and was highest in WildChat, although persistence alone did not determine the composite index.

### 4.3 Distribution and component structure of CBII

The distributional analysis evaluates whether the descriptive differences correspond to broad separation or are driven only by mean values. The density profiles in Figure 2A indicate a corpus-level shift rather than an isolated central-tendency effect: WildChat is displaced toward higher CBII values, Chatbot Arena is concentrated in the lower range, and LMSYS-Chat-1M occupies an intermediate position with a shape closer to WildChat. This pattern supports the interpretation that the datasets differ in their conversational structure, while preserving substantial overlap among individual conversations.

**Figure 2:**
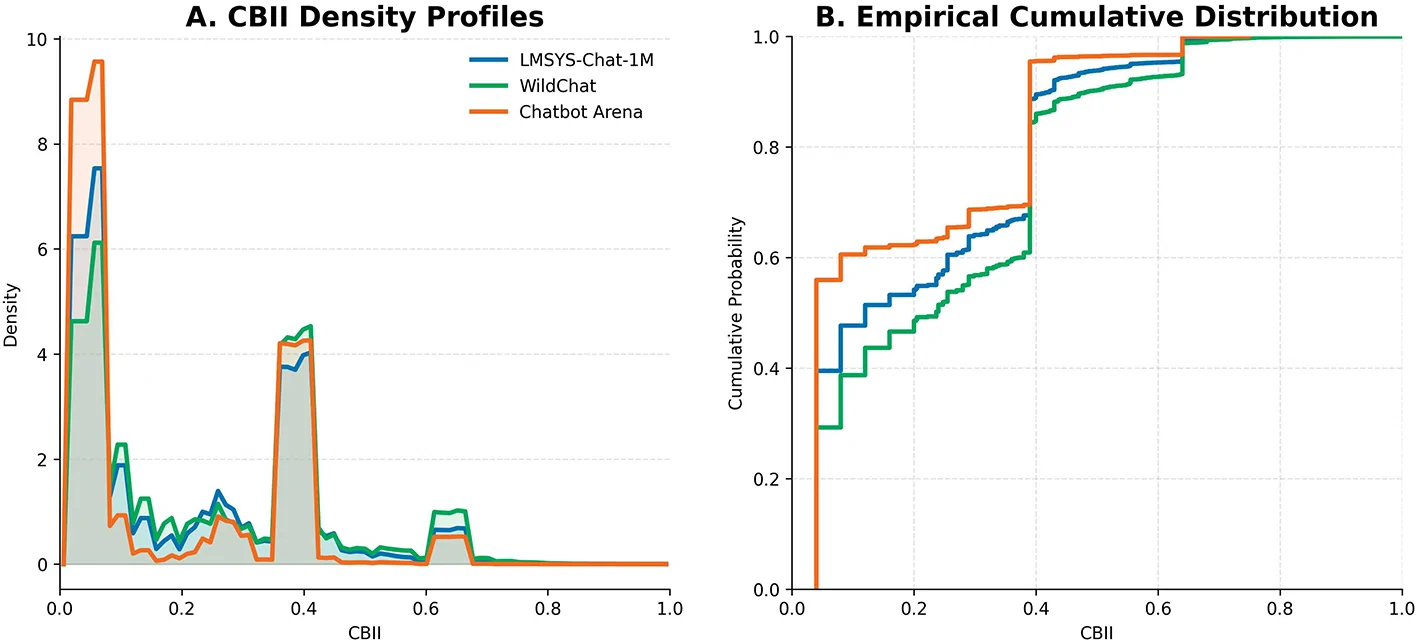
Distributional structure of CBII by dataset. **(A)** Smoothed density profiles. **(B)** Empirical cumulative distributions.

The breadth of the distributions in Figure 2A is analytically relevant because it prevents a deterministic reading of dataset membership. CBII varies widely inside each corpus, which means that the index summarizes heterogeneous structural and lexical configurations rather than assigning a fixed behavioral profile to a platform. The upward displacement of WildChat should therefore be interpreted as a larger proportion of conversations with higher combined structural and lexical indicator values, not as a categorical separation from LMSYS-Chat-1M or Chatbot Arena.

The empirical cumulative curves in Figure 2B strengthen this interpretation by showing where the separation occurs along the score range. WildChat accumulates probability more slowly at low CBII values, indicating that a larger fraction of its conversations occupies the middle and upper portions of the index. Chatbot Arena accumulates earlier, consistent with shorter comparison-oriented exchanges. LMSYS-Chat-1M remains intermediate across most of the curve, which supports a graded corpus-level ordering instead of a binary distinction between datasets.

The largest structural contrast is observed in Chatbot Arena. Although delegation-related markers remain relatively stable across datasets (CDR = 0.3168), persistence (IPF = 0.1190) and refinement-related markers (SRS = 0.0709) are substantially lower than in LMSYS-Chat-1M and WildChat. As a result, the reduction in CBII is primarily associated with lower persistence and fewer refinement-related lexical signals rather than with the absence of explicit task-request markers.

A comparison of component magnitudes further indicates that CDR has the highest average contribution among the three operational components. Delegation-related lexical markers consistently exceed both persistence and refinement-related markers, suggesting that explicit task-request language is the most frequently captured signal in the analyzed conversations. In contrast, SRS remains the lowest component across all datasets, indicating that revision-related lexical markers are less frequent than direct request markers. This component hierarchy is preserved across corpora despite differences in overall CBII levels.

### 4.4 Language control and pairwise effect sizes

Table 3 reports the language-control comparison used to evaluate dictionary-coverage bias. English accounts for 78.29% of LMSYS-Chat-1M, 57.68% of WildChat, and 88.51% of Chatbot Arena, while Spanish represents 2.39%, 2.48%, and 1.75%, respectively. Despite this unequal coverage, the CBII ranking remains stable in the global, English-only, and English + Spanish subsets. This result is important because CDR and SRS rely on English and Spanish lexical markers; therefore, the table evaluates whether the main ordering is preserved within the covered language space rather than assuming multilingual equivalence.

**Table 3:** Language-control comparison of mean CBII.

Dataset	English (%)	Spanish (%)	Global CBII	English-only	Eng. + Span.
LMSYS-Chat-1M	78.29	2.39	0.2123	0.2370	0.2361
WildChat	57.68	2.48	0.2463	0.3318	0.3288
Chatbot Arena	88.51	1.75	0.1762	0.1877	0.1872

The language-restricted estimates in Figure 3A provide a direct visual test of whether the CBII ordering is an artifact of dictionary coverage. The ordering is preserved in the global, English-only, and English + Spanish subsets, which indicates that the cross-corpus pattern is not produced by a single language composition. At the same time, the stronger English-only increase in WildChat indicates that dictionary sensitivity is not uniform across datasets. Figure 3B defines the boundary of this evidence: English coverage is uneven and Spanish coverage is small, so the control supports the comparative ranking while limiting interpretation to the languages explicitly covered by the marker dictionaries.

**Figure 3:**
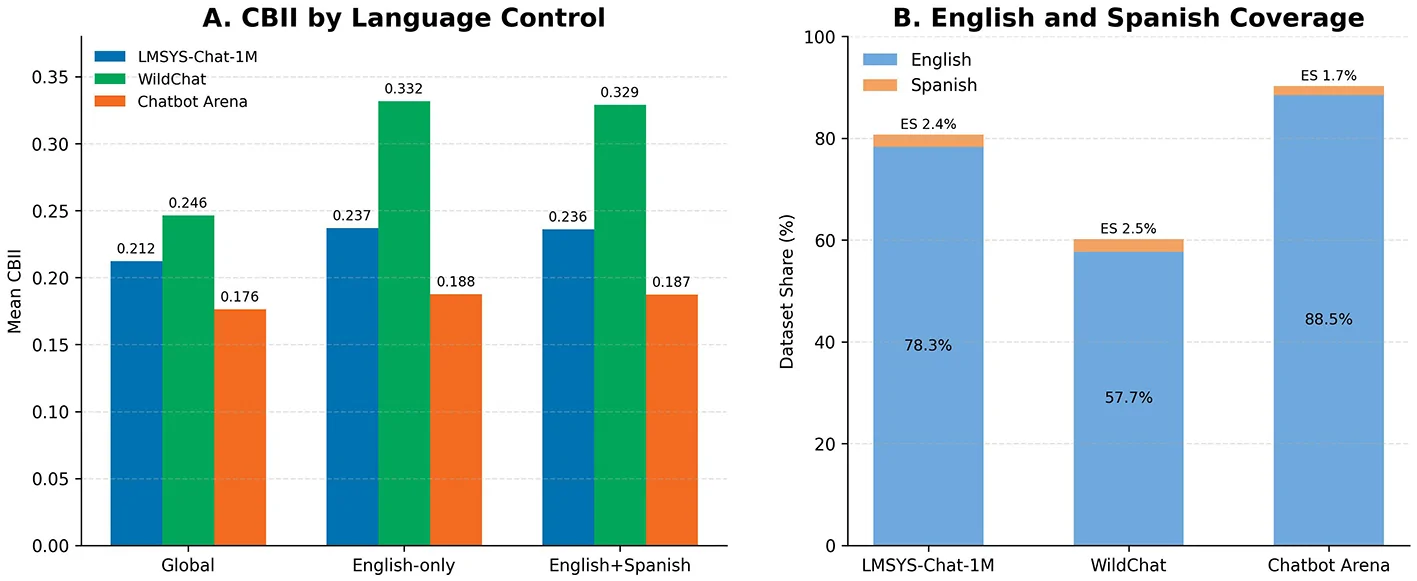
Language-control analysis. **(A)** Mean CBII under global, English-only, and English + Spanish subsets. **(B)** English and Spanish coverage by dataset.

Table 4 qualifies the practical magnitude of the observed differences through pairwise mean differences, percentile-based 95% bootstrap confidence intervals, Cohen's *d*, and Cliff's delta. Given the very large sample size, magnitude is more informative than null-hypothesis significance testing. The largest mean difference was observed between WildChat and Chatbot Arena [0.0701; 95% bootstrap CI (0.0698, 0.0746)], with a small-to-moderate standardized effect (Cohen's *d* = 0.363; Cliff's delta = 0.252). The contrasts involving LMSYS-Chat-1M were smaller, indicating that the corpus-level ordering is stable but not equivalent to a large separation between all datasets.

**Table 4:** Pairwise CBII effect sizes and 95% bootstrap confidence intervals.

Comparison	Mean diff.	95% CI	Cohen's *d*	Cliff's delta
LMSYS vs. WildChat	–0.0340	[–0.0363, –0.0318]	–0.18	–0.111
LMSYS vs. Arena	0.0361	[0.0338, 0.0384]	0.195	0.137
WildChat vs. Arena	0.0701	[0.0676, 0.0725]	0.363	0.253

### 4.5 Robustness and methodological controls

Sensitivity assessment examined whether the empirical ordering depends on the proposed aggregation scheme. Replacing the weighted index with *CBII*^*equal*^ produced highly consistent results across the analyzed datasets: WildChat remained highest, LMSYS-Chat-1M remained intermediate, and Chatbot Arena remained lowest. The agreement between both formulations was also high (Pearson = 0.9914; Spearman = 0.9876), indicating that the ranking is not a byproduct of assigning greater weight to persistence or delegation-related markers.

The IPF-threshold analysis in Figure 4A positions persistence saturation as a calibration parameter rather than as the determinant of the empirical result. Lowering the threshold from 10 to 5 user turns raises the absolute index level in all datasets, whereas increasing it to 20 lowers the index because persistence saturates later. The substantive evidence is the absence of ordinal instability under this rescaling: the three trajectories remain separated and preserve the same dataset order across thresholds. Thus, the cross-corpus result is robust to the selected persistence cutoff, while the absolute CBII level remains threshold-dependent. Figure 4B adds a magnitude-based reading of the same robustness claim. The bootstrap intervals indicate stable pairwise directionality, but the standardized effects remain small to moderate. This combination supports a cautious interpretation: CBII detects reproducible corpus-level differences, but those differences should be treated as structured shifts in observable behavior rather than as large separations between user populations.

**Figure 4:**
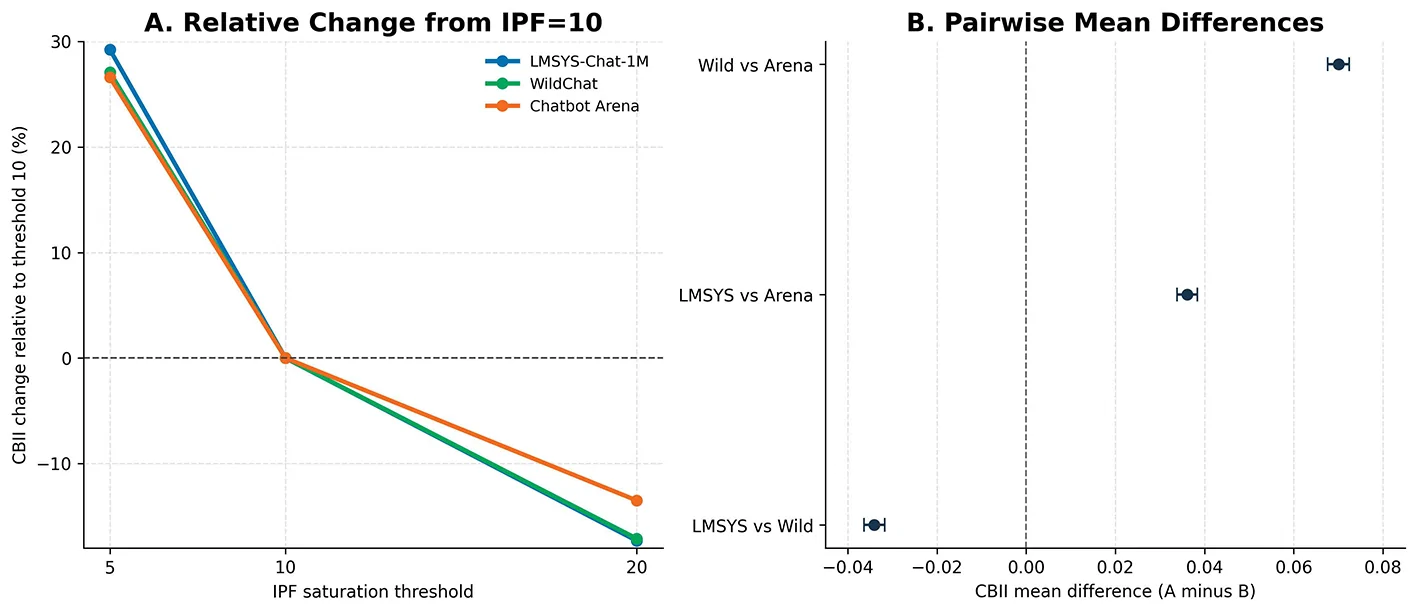
Robustness of CBII comparisons. **(A)** Relative change in mean CBII when the IPF saturation threshold is changed from the baseline of 10 user turns to 5 or 20 user turns. **(B)** Pairwise mean differences with 95% bootstrap confidence intervals.

Table 5 quantifies the main sources of structural non-equivalence among datasets. The IPF sensitivity control produced no ranking changes across thresholds 5, 10, and 20. Chatbot Arena retained 32,980 question-level analytical records and a mean CBII of 0.1763, nearly identical to the A/B-derived value but more appropriate for user-side interpretation. The single-turn control indicates that short structures are common in all datasets and especially dominant in Chatbot Arena. Exact first-prompt hashing further indicates minimal LMSYS–WildChat and WildChat–Arena overlap, but higher LMSYS–Arena overlap, consistent with their shared evaluation ecosystem.

**Table 5:** Quantitative methodological controls.

Control	Metric	Dataset/comparison	Value
IPF sensitivity	Thresholds tested	All datasets	5, 10, 20
IPF sensitivity	Ranking changes	All datasets	0
Arena question-level analysis	Question-level analytical records	Chatbot Arena	32,980
Arena question-level analysis	Question-level mean CBII	Chatbot Arena	0.1763
Single-turn structure	Single user-turn rate	LMSYS-Chat-1M	66.29%
Single-turn structure	Single user-turn rate	WildChat	60.16%
Single-turn structure	Single user-turn rate	Chatbot Arena	88.27%
Prompt overlap	Jaccard index	LMSYS–WildChat	0.0024
Prompt overlap	Jaccard index	LMSYS–Arena	0.0364
Prompt overlap	Jaccard index	WildChat–Arena	0.0004

Figure 5A shows the prevalence of component scores at or above the descriptive 0.5 cutoff. The concentration of high CDR rates across the three datasets indicates that explicit task-request markers are the most common high component-score signal represented by the framework, whereas high SRS rates are comparatively rare and high IPF rates depend more strongly on conversational length. This decomposition is necessary because similar CBII values can emerge from different operational configurations. Figure 5B connects this component structure with dataset architecture: Chatbot Arena combines the highest single user-turn rate with the lowest mean CBII, whereas WildChat combines the lowest single-turn rate with the highest mean CBII. The figure therefore supports a methodological interpretation of the Arena results: its lower index values are partly shaped by the paired-comparison format, not only by reduced delegation-related or refinement-related lexical signals. This panel provides a descriptive comparison of dataset structure and is not intended to establish an inferential association between single-turn prevalence and mean CBII.

**Figure 5:**
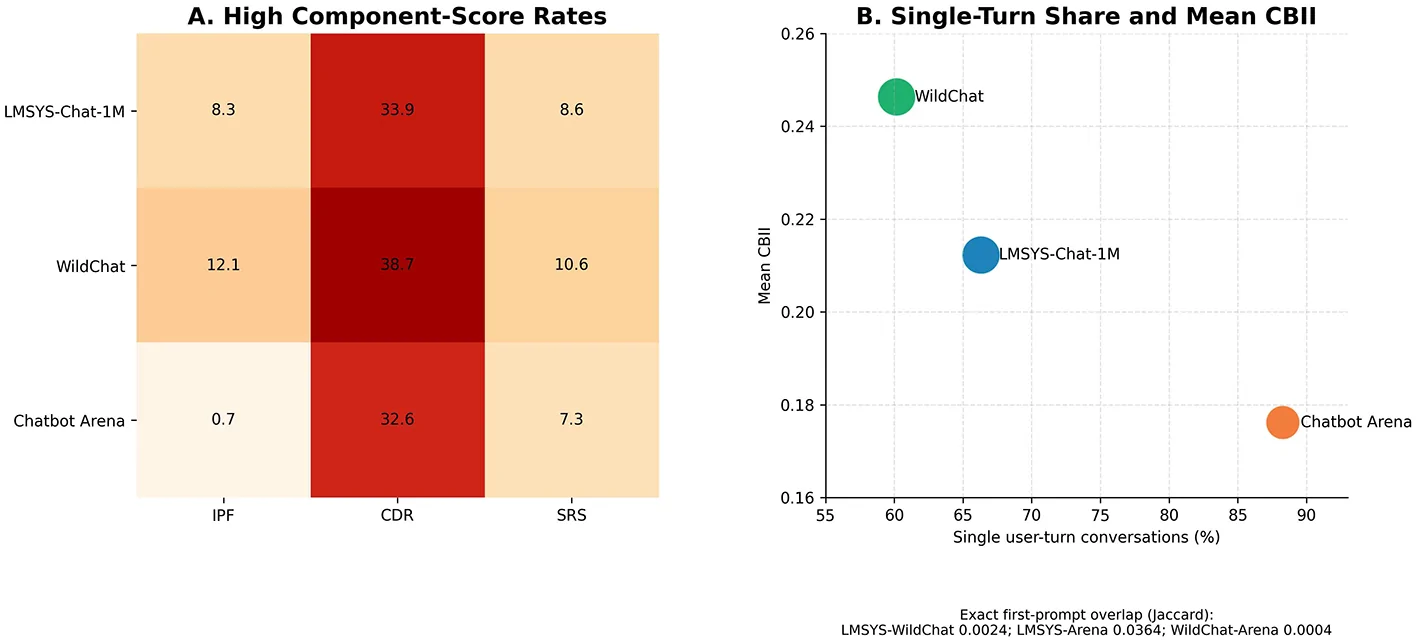
Component and structural controls. **(A)** Heatmap of high component-score rates (≥0.5). **(B)** Relation between single user-turn prevalence and mean CBII, with exact first-prompt overlap summarized below the panel.

Internal-structure correlations were retained as diagnostics rather than independent validation evidence because CBII is mathematically constructed from IPF, CDR, and SRS. At the global level, IPF–CBII remained low to moderate (Pearson = 0.2671; Spearman = 0.2876), whereas CDR–CBII was stronger (Pearson = 0.8434; Spearman = 0.8457). This pattern indicates that CDR shows the strongest internal association with the constructed composite index, while IPF and SRS provide complementary structural and lexical information.

## 5 Discussion

The results situate CBII within a recent debate on human–AI interaction that is no longer limited to accuracy or utility, but also examines how users adjust their behavior in response to generative systems. Evidence on personalized persuasion indicates that these models can adapt messages to individual profiles and modify beliefs with high communicative effectiveness (Matz et al., 2024; Teeny and Matz, 2024). Similarly, dialogues with AI have demonstrated the capacity to alter persistent beliefs in sensitive contexts, such as conspiracy theories (Costello et al., 2024), while education, health, and scientific writing document operational benefits together with risks of uncritical use, hallucinations, and displacement of expert judgment (Fütterer et al., 2023; Azamfirei et al., 2023; Haltaufderheide and Ranisch, 2024). These studies clarify important outcomes of human–AI interaction, but they do not provide a lightweight corpus-level measurement layer for comparing structural persistence, explicit request language, and revision-related lexical signals across open datasets. CBII addresses this narrower methodological gap: it does not assess persuasion, truth, clinical harm, or user dependence; it characterizes reproducible conversation-level signals that can guide subsequent semantic or human-coded analysis.

The methodological process indicates that the complete conversation is a suitable unit for studying observable behavioral intensity because persistence, delegation-related markers, and refinement-related markers are distributed across turns rather than isolated from dataset structure. LMSYS-Chat-1M and WildChat preserve sufficient structure to reconstruct user and assistant turns, while Chatbot Arena requires a more restrictive interpretation due to its A/B design. The question-level analysis indicates that Arena's mean CBII remains almost unchanged when prompts are no longer duplicated across response candidates, but its analytical role changes: it should be read as a conditional comparator, not as an independent corpus equivalent to LMSYS-Chat-1M or WildChat.

The quantitative results support the framework's usefulness as a descriptive audit layer. The main comparison rests on LMSYS-Chat-1M and WildChat as primary conversational corpora, with Chatbot Arena used to test how the index behaves under a structurally different A/B evaluation format. The stability of the ranking with *CBII*_*equal*_, English-only and English + Spanish controls, and IPF thresholds of 5, 10, and 20 indicates that the conclusion is not critically dependent on one weighting scheme, one language subset, or one persistence threshold. Pairwise effect sizes further temper the interpretation: the differences are systematic, but their magnitude is small to moderate rather than large.

The component-level results clarify the methodological role of IPF, CDR, and SRS. IPF captures sustained participation through user turns, making it useful for identifying longer conversational structures; however, it is sensitive to platform format and cannot be interpreted as reliance or dependence. CDR contributes more strongly to variation within the constructed composite because explicit task-request markers are frequent across the corpora, but CDR represents lexical evidence of request formulation, not confirmed transfer of responsibility or task completion. SRS contributes a lower average score because revision-related markers are less frequent and can occur in first-turn prompts; therefore, it should be read as a rule-based refinement marker rather than direct evidence of sequence-based self-correction. This component-level interpretation prevents any single indicator from being treated as a proxy for psychological reliance and reinforces the need to interpret IPF, CDR, and SRS as complementary operational signals.

Beyond the specific values obtained in each dataset, the main methodological contribution of this study is to demonstrate that reproducible indicators of human–AI interaction can be constructed from basic conversational information. In an environment where much current analysis relies on additional models, embeddings, or complex annotation procedures, CBII provides a transparent and auditable descriptive layer whose calculation can be inspected directly. This characteristic promotes replicability across studies and facilitates its use as a preliminary screening layer before richer semantic, temporal, or human-coded analyses are undertaken.

The limitations are substantial and define the appropriate scope of interpretation. CDR and SRS depend on English and Spanish dictionaries; they may omit indirect formulations, capture false positives, or underrepresent other languages. The language-control analysis reduces this concern for English and English + Spanish subsets, but it does not validate the dictionaries across the full multilingual space. IPF uses a saturation threshold of 10 turns, which is interpretable but conventional; the threshold analysis supports ranking stability without making the cutoff definitive. CBII combines components using theoretical weights, although equal-weight sensitivity reduces this risk. The datasets also contain platform, model, language, moderation, and single-turn biases, and Chatbot Arena remains structurally conditional because of its A/B comparison design. CBII therefore does not measure individual psychological dependence, addiction, or causal impact. It aggregates observable structural and lexical signals derived from secondary data and should be limited to descriptive corpus comparison.

## 6 Conclusions

This work proposes CBII as a transparent corpus-level framework for characterizing behavioral intensity in human–AI conversations. Its contribution is methodological: heterogeneous conversational records are converted into three auditable components—interaction persistence, delegation-related lexical markers, and refinement-related lexical markers—and then into a bounded composite index. The approach avoids trained classifiers, embeddings, or *post hoc* semantic labels, which makes each score traceable to explicit structural rules and dictionary matches.

Empirically, LMSYS-Chat-1M and WildChat functioned as the two primary conversational corpora, while Chatbot Arena served as a structurally conditional A/B comparator. WildChat consistently showed the highest mean CBII, LMSYS-Chat-1M occupied an intermediate position, and Chatbot Arena obtained the lowest value. This ordering was preserved under equal weighting, language controls, IPF threshold sensitivity, and question-level Arena analysis. Effect-size estimates indicate that these differences represent reproducible corpus-level shifts, but not large separations between user populations.

The main limitations are tied to the deliberately lightweight design. IPF depends on a conventional saturation threshold, CDR and SRS depend on English and Spanish lexical dictionaries, and SRS cannot by itself prove sequence-based refinement because revision markers may occur in first-turn prompts. Future work should validate the marker dictionaries with human annotation, expand them across languages and pragmatic styles, and test the framework in domain-specific corpora such as programming, education, healthcare, and emotional support. CBII should therefore be used as a first-layer descriptive audit, not as an individual diagnostic scale, causal measure, or validated psychological reliance instrument.

## Data Availability

Publicly available datasets were analyzed in this study. The datasets analyzed in this study are publicly available from the following repositories: LMSYS-Chat-1M (Hugging Face): https://huggingface.co/datasets/lmsys/lmsys-chat-1m, WildChat (Hugging Face): https://huggingface.co/datasets/allenai/WildChat, Chatbot Arena Conversations (Hugging Face): https://huggingface.co/datasets/lmsys/chatbot_arena_conversations. To support peer review and reproducibility, the minimal reproducibility package is publicly available at https://github.com/wvillegas01/CBII-reproducibility-package.
